# Cardiovascular health technology assessment: recommendations to improve the quality of evidence

**DOI:** 10.1136/openhrt-2018-000930

**Published:** 2019-02-27

**Authors:** Colin Berry, David Corcoran, Kenneth Mangion

**Affiliations:** 1 BHF Glasgow Cardiovascular Research Centre, Institute of Cardiovascular and Medical Sciences, University of Glasgow, Glasgow, UK; 2 West of Scotland Heart and Lung Centre, Golden Jubilee National Hospital, Clydebank, UK; 3 Institute of Cardiovascular and Medical Sciences, University of Glasgow, Glasgow, UK

**Keywords:** coronary artery disease, imaging and diagnostics, public health

## Abstract

The aim of this article is to review the role of Health Technology Assessment (HTA) organisations in appraising and recommending innovative cardiovascular technologies. We consider how bias impairs the quality of evidence from clinical trials involving cardiovascular healthcare technologies. Finally, we provide recommendations to HTA organisations to take account of bias when making guideline recommendations.

Clinical research studies of medical devices, diagnostics and interventions in cardiovascular healthcare are susceptible to impairment through bias. While HTA organisations, such as the National Institute of Health and Care Excellence, may require reviewers to take account of bias, there are uncertainties as to how this is achieved, especially in cardiovascular technology trials. This becomes more relevant given that large trials are few in number; therefore, the quality of evidence from an individual trial may have a large bearing on guideline recommendations and clinical practice.

HTA organisations should drive improvements in the design and rigour of randomised trials. The evolving landscape of cardiovascular healthcare technologies and related trials presents a challenge for HTA organisations and healthcare providers. The rapid turnover of evidence is externally relevant because the period from the trial publication to implementation of HTA guideline recommendations by healthcare providers may be prolonged, by which time new evidence may have emerged from subsequent trials. Implementation of a cardiovascular healthcare technology including be it a medical device, diagnostic or intervention may have profound implications for healthcare providers. These technologies may have high absolute costs and access may be influenced by socioeconomic and geographic factors.

## Introduction

Innovative technologies drive advances in cardiovascular healthcare. Novel technologies may target a new indication, a gap in healthcare or simply improve the standard of care. Beyond medical altruism and human invention, technological advances are energised by commercial factors. Ultimately, service user access to new technologies, such as CT coronary angiography, is multifactorial, being influenced by infrastructure, funding streams and local expertise. This is especially the case in cardiovascular healthcare. Health Technology Assessment (HTA) varies between countries. HTA organisations may be nonexistent. There may or may not be a legislative requirement for HTA reviews to be considered and/or used by government in decision-making for healthcare. A summary of HTA organisation by country is shown in table 1.

**Table 1:** WHO Health Technology Assessment by country

Country	Governance		Purpose	Process transparency			Public communication
	National HTA organisation	Legislative requirement	HTA used in decision-making	Guidelines	Conflict of interest declaration	Civil society participation	Reports public available
Afghanistan, Albania, Azerbaijan, Barbados, Bulgaria, Bahrain, Cambodia, Central African Republic, Comoros, Côte d'Ivoire, Democratic Republic of the Congo, Eritrea, Fiji, Micronesia (Federated States), Ghana, Kenya, Kiribati, Lao People's Democratic Republic, Lebanon, Madagascar, Sri Lanka, Libya, Republic of Moldova, Macedonia, Maldives, Peru, Qatar, San Marino, Somalia, Vietnam	No/unsure	No/unsure	No/unsure	No/unsure	No/unsure	No/unsure	No/unsure
Armenia, Australia, Brazil, Canada, Finland, Guatemala	Yes	Yes	Advisory	Yes	Yes	Yes	Yes
Austria, Belgium	Yes	No	Advisory	Yes	Yes	No	Yes
Bangladesh	No	Yes	Unsure	Yes	No	Yes	Yes
Belarus	Yes	No	Advisory	Yes	Yes	Yes	No
Benin, Denmark, Thailand, United Kingdom	Yes	No	Advisory	Yes	Yes	Yes	Yes
Bhutan	Yes	Yes	Advisory	Yes	Yes	No	No
Cape Verde	Yes	No	No	No	No	No	Yes
Cameroon, Monaco, Trinidad and Tobago	No	No	Advisory	No	Unsure	No	Unsure
China	Yes	No	Advisory	No	No	Unsure	No
Costa Rica	No	No	Advisory	No	Yes	Yes	Yes
Croatia	Yes	Yes	Advisory	Yes	Yes	Yes	Yes
Cuba	Yes	No	Advisory	No	No	No	Yes
Cyprus	Yes	No	Advisory	Unsure	Yes	No	No
Germany, Republic of Korea, Kazakhstan, Romania	Yes	Yes	Mandatory	Yes	Yes	Yes	Yes
Ecuador	Yes	No	Advisory	Yes	Yes	Unsure	Yes
Egypt	No	Yes	Advisory	No	Unsure	No	Yes
Estonia	Yes	Yes	Advisory	No	Yes	Unsure	Yes
Ethiopia	No	No	Mandatory	Unsure	No	Yes	Yes
Gambia, Slovenia, Sudan, Timor-Leste	No	Yes	Advisory	Unsure	Unsure	Unsure	Unsure
Georgia	Yes	Yes	Mandatory	Unsure	Unsure	Unsure	Unsure
Ghana	Yes	No	Advisory	Unsure	Unsure	Unsure	Unsure
Hungary, Iran	Yes	Yes	Advisory	Yes	No	Yes	No
Iceland, Montenegro	No	No	Advisory	Unsure	Unsure	No	Unsure
India	Yes	No	Advisory	Yes	Yes	Yes	No
Indonesia	Yes	No	Advisory	Unsure	Yes	Yes	Yes
Iraq	Yes	Unsure	Advisory	Yes	Unsure	Yes	Yes
Italy	Yes	No	Advisory	Yes	Yes	Yes	Yes
Jamaica	Yes	Unsure	Advisory	Yes	Unsure	Yes	Unsure
Japan, Norway	Yes	No	Advisory	Yes	Yes	No	Yes
Jordan	Yes	Unsure	Advisory	Unsure	Unsure	Unsure	Unsure
Latvia	No	Yes	Mandatory	Yes	No	Yes	Unsure
Lithuania	Yes	Yes	Advisory	No	No	No	No
Luxembourg	Yes	Yes	Advisory	Unsure	Yes	No	Yes
Malaysia	Yes	No	Advisory	No	Yes	No	No
Mali	Yes	Yes	Mandatory	Yes	No	Yes	Yes
Malta	Yes	Yes	Advisory	No	Yes	Yes	No
Mexico	Yes	No	Advisory	Yes	Yes	No	Yes
Mozambique	Yes	No	Unsure	No	No	No	Yes
Nauru	No	No	Mandatory	No	No	Yes	Yes
Nepal	No	No	Unsure	No	No	Yes	Yes
Netherlands	Yes	Yes	Unsure	Yes	Yes	Yes	Yes
New Zealand	Yes	No	Advisory	Unsure	Yes	Yes	Yes
Philippines	No	Yes	Advisory	No	Yes	No	No
Poland, Portugal	Yes	No	Advisory	Yes	Yes	Yes	No
New Zealand	Yes	No	Advisory	Unsure	Yes	Yes	Yes
Russian Federation	No	No	Unsure	Yes	Unsure	Yes	No
Saudi Arabia	Yes	Yes	Advisory	Yes	Yes	Yes	No
Serbia	Yes	Yes	Mandatory	No	Unsure	Yes	Yes
Singapore	Yes	Yes	Advisory	No	Yes	No	Yes
South Africa	No	Yes	Advisory	Yes	Yes	Yes	Yes
Spain	Yes	Yes	Advisory	Yes	Yes	No	Yes
Saint Vincent and the Grenadines	Yes	Yes	Advisory	Unsure	Unsure	Yes	Yes
Switzerland, Slovakia, Sweden	Yes	Yes	Advisory	Yes	Yes	No	Yes
Syrian Arab Republic	Yes	Yes	Advisory	Yes	Yes	No	No
Tuvalu	Yes	Yes	Advisory	No	No	Yes	Yes
Tanzania, United Republic of	No	Unsure	Advisory	Yes	Unsure	Yes	No
Turkey	Yes	No	Advisory	Yes	Yes	Yes	No
USA	Yes	No	Advisory	Yes	Yes	Yes	Yes

HTA, Health Technology Assessment.

Access to new healthcare technologies in the National Health Service (NHS) in England and Wales is guided by the National Institute of Health and Care Excellence (NICE). The Department of Health supports NICE and commissions guidance. NICE is highly valued by industry, healthcare providers and regulators worldwide. NICE inevitably has a role in promoting the adoption of new healthcare technologies.^1^ Here, we consider ‘realistic medicine’ in cardiovascular healthcare, that is, personalised care which is patient-centred and minimises unwarranted variation in practice and reduces harm and waste.^2 3^

We draw attention to the issue of bias within cardiovascular trials and highlight the role that NICE and other HTA organisations might adopt to drive improvements in the standards of trials and quality of evidence.

## NICE guidance for cardiovascular healthcare technologies

Groves *et al*^1^ review the role of NICE in promoting innovative cardiac technologies. The authors are leaders of the NICE Medical Technologies Evaluation Programme and Centre for Health Technology Evaluation. Their review presents an informative insight into how NICE evaluates medical technology for the cardiovascular system. Since 2009, 33 Medical Technology Guidance (MTG) documents have been issued, including 7 on medical technologies appraisals, 3 on diagnostics, and 23 on technology innovations relating to the cardiovascular system.

The Diagnostic Advisory Committee of NICE reviews diagnostic technology if there is a potential to improve health outcomes, even if there is a cost increase to the NHS. Specifically, MTGs are published for innovative technologies that have patient benefits and are associated with cost savings to the NHS. Interventional Procedures Guidance (IPG) summarises the safety and efficacy of innovative procedures. Since 2003, 73 IPGs have been published on the cardiovascular system, notably for Transcatheter Aortic Valve Implantation (IPG 586, July 2017). Technology appraisals are focused on potential health benefits and cost-effectiveness.

Effective, timely coordination between NICE programmes that work in parallel is important. By way of example, Groves *et al*^1^ cite the Diagnostic Guidance issued on a new generation of CT scanners for coronary artery imaging and the high-sensitivity troponin assays used for the early rule-out of non-ST segment elevation myocardial infarction. Both documents link contemporaneously to the NICE Clinical Guideline-95 (CG95)^4^ update on the assessment and diagnosis of chest pain. The MTG32 on fractional flow reserve-CT by HeartFlow was also timed to link with the CG95 update.^5^ These are examples of effective coordination between review groups within an HTA organisation.

## Wider implications of NICE for cardiovascular healthcare

NICE acts at the interface where innovative cardiovascular technologies meet the NHS. The healthcare landscape is broad and complex, extending across primary and secondary care, academia, industry, government, patients and the public, and realistic medicine is increasingly advocated.^2 3^

Key strengths of the NICE approach include involvement of experts from relevant disciplines and engagement with stakeholders including professional societies, industry and the public. However, the variable quality of evidence, for example, from open-label trials, advances in technology and turnover of trials, present key challenges for HTA organisations when developing guidance on cardiovascular healthcare technologies. Open-label trials are the norm opening the door to bias and impaired clinical evidence. Blinded, sham (placebo)-controlled trials of technologies (or linked strategies) are rare but all the more impactful.^6 7^ By contrast, a randomised, double-blinded, placebo-controlled design is the established norm for evaluating medicines. This disparity inevitably reduces the quality of evidence for cardiovascular technologies compared with medicines and becomes problematic when the quality of the evidence is discounted by HTA organisations and practice guideline committees. For example, the classes of guideline recommendations (I–III) and levels of evidence (A–C) are directly influenced by the number of trials but calibration based on the quality of evidence is unclear.^8^

The regulatory approval process for medical devices and drugs differs. In Europe, medicines are overseen by the European Medicines Agency, which requires evidence of clinical effectiveness and safety before regulatory approval. There is no single agency designated for licensing devices in Europe.^9^ In fact, multiple agencies may provide (or reject) approvals for device use, meaning a device may be endorsed by one agency and not by another. Weaker forms of evidence seem to be sufficient for approval of medical technologies, including diagnostics and implantable devices. One case in point is the Nanostim leadless pacemaker which was licensed based on 90-day follow-up information in 30 patients. The device was granted a Conformité Européenne (CE) mark by the British Notified Body, British Standards Institution, whereas the German notified body turned down the application. In 2016, St Jude Medical halted the availability of the device because of battery issues.^10^ However, a lack of postmarketing safety information has raised concerns about this device and the regulatory process overall. Furthermore, there is no unifying registry for postmarketing reporting of adverse events. A test or device may inherit approval status related to historical products. One example of this is the transvaginal mesh which was marketed in Europe and the USA using equivalence data, however, issues with the new device emerged in relation to unexpected side effects.^11 12^

## Quality of evidence: should HTA organisations be a driver for change?

### Internal validity and the risk of bias

Clinical trials of medical devices, diagnostics and interventions are susceptible to bias, more so than placebo-controlled trials of new medicines. Open-label technology trials are the norm mainly due to logistical and/or cost reasons, meaning the investigators, clinicians and patients are unblinded. Unlike in trials of cardiovascular medicines, active comparators for imaging interventions, for example, CT coronary angiography, may not be adopted on grounds of cost and logistics. A cardiovascular intervention may have an obvious effect, for example, surgical scar, limiting blinding. Cardiovascular healthcare is a specialised field and professional bias may be operative. For example, a clinician who is trained and employed to undertake cardiovascular imaging may be susceptible to overt or subconscious bias if s/he is an investigator in a trial of an imaging-based intervention versus an alternative non-imaging intervention. Commercial influences may also be relevant relating to reimbursements (in-kind or direct) to the provider (physician, hospital or sponsor) for the use of a device for example, coronary stent.

The guidance issued by HTA organisations is a function of the available clinical evidence, the quality of the evidence, its cost-effectiveness and the overall approach of the organisation to developing the guidance (table 1). In decision-making, HTA committees may take account of evidence quality and quantity as well as sources of bias and whether or not interventions were blinded to the operator, the patient and the assessor. Bias may not be obvious and so difficult to identify and quantify. Nonetheless, we are unclear about how bias is quantified and whether there is a standardised approach to qualify the strength of the recommendation according to the quality of clinical evidence. NICE periodically updates its methods and in doing so is guided by the developing thinking of research designers and HTA methodologists who work with NICE and stakeholders’ comments.

## Examples of potential bias in cardiovascular technology trials

Bias impairs the quality of the evidence in several ways.^13^ Types of bias in clinical trials are summarised in table 2. Clinical evidence that is demeaned by bias will have an undesirable impact on society. Consider a large, multicentre, randomised controlled trial. Is this synonymous with high-quality evidence? We think not, and this is especially relevant to cardiovascular technologies. There are multiple mechanisms by which bias impairs the quality of evidence from a randomised trial of a cardiovascular healthcare technology. Allocation concealment is commonly not implemented in technology trials on grounds of logistics and costs. In an open-label trial, the study procedures and standard care management become susceptible to the effects of overt and unmeasured bias. The investigators may have professional interests in the intervention, for example, radiologists participating as investigators in a trial of two clinical strategies, one with imaging and the other without. This scenario raises the potential of professional and academic bias. In a strategy trial, clinician enthusiasm to gain access to a new technology (the intervention) may reduce the quality of standard of care in the control group. Another scenario is where the eligibility criteria of a trial are designed to enrich it with ‘responders’, rendering the trial population less representative of all-comers and reducing external validity.

**Table 2:** Potential sources of bias in clinical trials

Bias type	Definition and examples	Identifiable	Quantifiable
Academic bias	The investigators leading study are advocates for the intervention.	Y	Y
Ascertainment bias	Un-blinded study design in which the outcome evaluations are susceptible to unmasked observer detection bias. Open-label studies, such as imaging and device trials (without a sham) are susceptible to ascertainment bias	Y	Y/N
Comparison group bias	If incorrect control/sham group is chosen, the intervention may appear to be more, or less, effective	Y	N
Fraud bias	Intentional fraud (rare)	Y	Y
Funding availability bias	Focus of studies on questions more readily funded (commercial interest)	Y/N	Y/N
Hidden agenda bias	Study designed to demonstrate a prerequired answer.	Y/N	Y/N
Intervention bias	Effects of a learning curve when investigating a new technology	Y/N	Y/N
Measurement bias	Measurement influences the respondent’s behaviour and responses, reflecting ‘response shift’ and relatedly a Hawthorne effect. This becomes relevant if there is an interaction between the intervention and the measurement tool (eg, a training effect)	Y/N	Y/N
Observer bias	Patients allocated to treatment arm followed more intensely/more favourably	Y/N	Y/N
Publication bias	Positive results are more likely to get published	Y	Y/N
Regulation bias	Overly restrictive or permissive review boards confounding the path to first-patient in	Y/N	Y/N
Sample choice bias	Exclusion of minority groups (recruitment bias), older groups (age bias) and women (sex bias)	Y	Y/N
Selection bias	Exclusion of potentially eligible patients	Y	Y/N
Selective reporting bias	Selective reporting of positive results	Y	Y
Withdrawal bias	Handling missing data: Are the number of withdrawals and their reasons stated in the report? Are the number of withdrawals similar in each of the groups, or not? Is the overall number of withdrawals comparable to the number of patients that contribute to a difference in the primary outcome?	Y	Y/
Wrong design bias	Incorrect study design to answer a question (eg, a randomised study rather than post-approval outcome research)	Y	Y/N

Consider a novel diagnostic test being assessed for noninferiority versus the standard care test. By design, the likelihood of noninferiority criteria being met is enhanced if the trial is enriched with patients with mild disease (low adverse event rate) which, in turn, increases the true (actual) noninferiority margins compared with the predicted margin. Withdrawal rates, potentially of non-responders, may be appreciable. For these reasons, the per-protocol analysis of a noninferiority trial has key importance. If the primary outcome is linked to the randomised strategy (eg, coronary revascularisation in a trial of two invasive tests of coronary stenosis severity, and the decision to perform revascularisation is at the discretion of an unblinded investigator), then the primary outcome becomes highly susceptible to ascertainment bias.

The timing of randomisation is relevant.^14^ If the intervention (eg, an imaging scan) is performed after randomisation, and if the randomised assignment was known to the imaging staff performing the scan, scan quality and compliance may be affected. In a trial without allocation concealment, administration of the interventions may be susceptible to bias. For example, in a comparative effectiveness trial of an imaging intervention (eg, CT coronary angiography), the protocol may involve local site reads and local decisions; however, the trial may also include checks of the imaging scans by the lead site (for quality assurance and compliance with protocol) but without similar checks of the quality of the intervention in the standard care group (eg, treadmill exercise testing, invasive coronary angiography). Standard care medications may be affected, such as enhanced treatment in the active group and/or diminished treatment in the control group. Prescription of evidence-based medicines or procedures may be less than expected for the level of risk or disease burden in the control group. Follow-up contacts by the investigators may be influenced by group assignment for example, more frequent patient contacts (documented or not) in the active group compared with the control group. Therefore, when considering the results of a randomised controlled trial, are the results achieved the direct consequences of the intervention, an increase in standard care in the active group, a reduction in standard care in the control group or a combination of these factors? The answer may be difficult, if not impossible, to quantify. Bias may be imperceptible and difficult to quantify, but this is no reason for neglect. One example is measurement and reactivity bias (table 2). The Measurement Reactions in Trials study has been commissioned by the Medical Research Council/National Institute of Health Research Methodology Research Programme to develop expert guidance on how to avoid bias due to measurement reactivity in randomised trials of interventions to improve health.^15^

Academic and commercial conflicts of interest may influence the design and conduct of trials involving healthcare technologies. If the sponsor of the trial also funds the trial and owns the technology (an industry-sponsored study), then academic and commercial interests are linked. It is not in the commercial interest of a company, or investigators with proprietary or professional interests, to deliver a ‘negative trial’; therefore, a critical appraisal of the design of the trial becomes important when considering the quality of the evidence, and related practice guideline recommendations.

### Examples of guideline-changing trials that were susceptible to bias

The Scottish COmputed Tomography of the HEART (SCOT-HEART) was an open-label clinical trial that took place in chest pain clinics in NHS Scotland. Standard care was guideline-directed patient management mainly based on treadmill exercise testing. The intervention was standard care guided by an additional CT coronary angiogram. The control group followed standard care but there was no additional test (active or sham). The intervention was randomly allocated 1:1 in 4146 participants with stable chest pain. The trial had an open-label design and the intervention was unmasked. The primary endpoint of the study was the proportion of patients diagnosed with angina pectoris secondary to coronary heart disease at 6 weeks. The clinicians who determined the primary outcome were familiar with the treatment group of the participants.^16^ SCOT-HEART was a ‘positive’ trial in favour of the CT-guided strategy but by design the primary outcome was therefore susceptible to bias. On the other hand, unexpectedly, symptoms and quality of life (secondary outcomes) improved less with the CT-guided strategy as compared with standard care. Nonetheless, the updated NICE-95 clinical guidelines recommended CT coronary angiography as the first-line test for patients with stable chest pain. The CG95 guideline update issued in November 2016 did not mention the Clinical Evaluation of Magnetic Resonance Imaging in Coronary heart disease 2 trial that had been published in September 2016. This trial presents evidence in favour of stress imaging over CT coronary angiography^17^ and highlights the importance of updates that take account of new evidence. A summary of the major published and ongoing diagnostic strategy trials in patients with suspected ischaemic heart disease is shown in table 3.

**Table 3:** Focus topic: contemporary multicentre strategy trials of cardiac imaging in patients with suspected stable IHD

Title	Design	Result
Published trials		
**PROMISE** NCT01174550	Aim: To compare CTCA versus standard care functional assessment Setting: Hospitals in the USA Sample size: 10 003 Primary endpoint: Death, MI, unstable angina	Compared with noninvasive functional testing, an initial strategy of CTCA did not improve clinical outcomes at a median follow-up of 2 years (primary endpoint event of 3.3% in the CTCA versus 3.0% in the noninvasive functional testing group)
**SCOT-HEART** NCT01149590	Aim: Comparison of usual care to CTCA Setting: Chest pain and cardiology clinics in hospitals in Scotland Sample size: 4146 Primary endpoint: Death from coronary heart disease or nonfatal myocardial infarction at 5 years	Th primary endpoint was lower in the CTA group than in the standard care group (2.3% [48 patients] vs 3.9% (81 patients); HR, 0.59; 95% CI, 0.41 to 0.84; p=0.004
**CE-MARC2** NCT01664858	Aim: To assess whether unnecessary invasive coronary angiograms are reduced using stress perfusion CMR at 3.0 Tesla versus MPS versus NICE guideline-directed care Setting: Chest pain and cardiology clinics in hospitals in the UK Sample size: 1202 Primary endpoint: Unnecessary invasive coronary angiography occurring within 12 months in each arm	The primary outcome occurred in 69 (28.8%) in the NICE guideline-directed group, and 36 (7.5%) and 34 (7.1%) in the CMR and MPS groups, respectively. There was a statistically significant lower adjusted OR of unnecessary angiography in the CMR versus NICE guideline-directed group (0.21, p<0.001), with no difference between the CMR or MPS group (1.27, p=0.32)
Trials yet to publish		
**ISCHEMIA** NCT01471522	Aim: To assess whether or not an initial invasive strategy of invasive angiography and optimal revascularisation if feasible, in addition to OMT in patients with stable CAD and at least moderate ischaemia on noninvasive ischaemia improves health outcomes compared with OMT alone Setting: Hospitals worldwide Sample size: 8000	Time to first occurrence of cardiovascular death or nonfatal myocardial infarction
**DISCHARGE** NCT02400229	Aim: To evaluate whether CTCA-based management over invasive coronary angiography-guided care is superior in patients with stable angina and an intermediate pretest probability (10%–60%) of CAD Setting: Hospitals in Europe Sample size: 3546	Cardiovascular death, nonfatal myocardial infarction and nonfatal stroke at a maximum follow-up of 4 years.
**FORECAST** NCT03187639	Aim: To assess whether routine FFR-CT is superior, in terms of resource utilisation, when compared with routine clinical pathway algorithms recommended by NICE Setting: Single centre in the UK Sample size: 1400	Resource utilisation at 9 months.
**MR-INFORM** NCT01236807	Aim: To assess whether or not stress perfusion CMR is noninferior to invasive coronary angiography and FFR measurement for the management of patients with angina Setting: Hospitals in the UK, Europe and Australia Sample size: 918	Death, myocardial infarction and repeat coronary revascularisation at 1 year
**CorCTCA** NCT03477890	Aim: To assess the prevalence of microvascular or vasospastic angina and the impact of invasive coronary artery function tests, in patients with nonobstructive CAD Setting: Three hospitals in the UK Sample size: 250	The between-group difference in the reclassification rate of the initial diagnosis following disclosure of invasive coronary artery function tests

CAD, coronary artery disease;CE-MARC2, Clinical Evaluation of Magnetic Resonance Imaging in Coronary heart disease 2; CMR, Cardiac Magnetic Resonance; CTCA, CT coronary angiography;FFR, fractional flow reserve;IHD, ischaemic heart disease; MI, myocardial infarction;MPS, myocardial perfusion scintigraphy; NICE, National Institute of Health and Care Excellence; OMT, optimised medical therapy; PROMISE, progesterone in recurrent miscarriage; SCOT-HEART, Scottish COmputed Tomography of the HEART.

In phase III and phase IV trials in interventional cardiology, coronary revascularisation is commonly included as a component of the primary outcome. This procedure is triggered by a medical decision and the primary outcome becomes susceptible to bias when the decision for revascularisation is made by an unblinded clinician who may also be an investigator in the trial. The design becomes all the more problematic if the revascularisation event is discounted within a time period when revascularisation might commonly occur, for example, 45 days^18^ or 60 days.^19 20^ The primary outcome can therefore be influenced by an investigator who may purposefully or subconsciously schedule a revascularisation procedure days before or after this cut-off.

### Value of a placebo or sham procedure to achieve allocation concealment

A placebo presents the means to mitigate bias.^21^ A matched-placebo should blind the patients, the investigators and the funders of the trial. A randomised, double-blind, placebo-controlled trial is the reference approach for assessing the effects of an intervention. A common alternative design is the use of active-controlled trials in cardiovascular populations, since a placebo may present unacceptable ethical implications in the face of withdrawal of standard care; for example, heart failure with reduced ejection fraction and treatment with an ACE inhibitor.^22^ Sham-controlled trials are rare in the cardiovascular space. The standards of clinical trials of healthcare technologies generally fall short of placebo-controlled trials of new medicines. We believe it is undesirable to have different standards for trials involving healthcare technologies as compared with those with medicines.

### How does NICE quantify bias in technology trials?

In the NICE manual for reviewers,^23^ a number of steps are advocated to take account of bias. Two reviewers should independently assess study and conference abstracts and report their quality. In order to reduce inter-rater variability, reviewers should assess clinical evidence using the Grading of Recommendations Assessment, Development and Evaluation (GRADE)^24^ and the Quality Assessment of Studies of Diagnostic Accuracy included in Systematic Reviews (QUADAS-2).^25^ QUADAS-2 is intended to assist systematic reviews of diagnostic accuracy studies. The GRADE approach is not necessarily a guideline for rating individual studies, rather the totality of information across several studies and/or systematic reviews.

The quality of a trial can be assessed using an instrument such as the Jadad scale.^26^ Although not without limitations,^27^ the use of such a tool presents an opportunity to objectively standardise trial evaluation. The Jadad scale involves blinded quality assessment of studies to identify observer bias, but its use may be limited by inter-rater variability and seems insensitive to the different forms of bias that may be operative in a trial (table 2). For these reasons, we advocate an improved and standardised approach to identify and quantify bias within a trial in order to calibrate and qualify the strength of related guideline recommendations (table 4). We are unclear how bias is identified and quantified by NICE reviewers when considering cardiovascular technology trials. This issue becomes all the more relevant given that large trials are few in number; therefore, the quality of evidence from an individual trial may have a large bearing on guideline recommendations.

**Table 4:** Quality of evidence assessment tool. (1) Is the study, a randomised, double-blind, placebo- (or sham-) controlled clinical trial? Yes / no. (2) considering the design of the trial, please answer the following questions:

	Question to respondent	Investigator or sponsor response	Reviewer response	Supporting evidence statement
	In your opinion, is the study associated with the following types of bias?	5-option response (strongly agree–strongly disagree) or not applicable	5-option response (strongly agree–strongly disagree) or not applicable	
1	Academic			
2	Ascertainment			
3	Comparison group			
4	Fraud and/or misconduct			
5	Funding availability			
6	Hidden agenda			
7	Intervention			
8	Measurement			
9	Observer			
10	Publication			
11	Regulation			
12	Sample choice			
13	Selection			
14	Selective reporting			
15	Withdrawal			
16	Wrong design			

Quality of evidence score = ________%. If the response to question 1 is ’yes’, then the initial quality of evidence score is 100%. If the response to question 1 is ’no’, then the score is <100%. Question 2 has 16 sub-questions, each with an ordinal response. An ordinal, monotonic scaling response (Likert scale) is proposed to rate the respondent’s perspective on evidence quality. Where the response relates to a binary state the extreme response would be expected. The response is weighted from 1 (no bias) to 5 (evidence of bias). The numeric responses should be added to give a summative score that should then be deducted from 100 and expressed as a percentage. The ordinal response informs the extent of impairment in the medical evidence relating to the study. Since accurate and precise measurement of bias may be difficult or impossible, the response is recorded using an ordinal scale. Sample choice bias may be rated based on evidence of exclusion of minority groups (recruitment bias), older groups (age bias) and women (sex bias), or, where there appears to be a clinically meaningful difference in the proportion of the trial population represented by this subgroup compared with the population prevalence.

We believe that HTA organisations and regulatory bodies should be drivers for improvement in the design of randomised trials of cardiovascular technologies. We call for practice guideline recommendations by HTA organisations to be weighted on the quality of the evidence, not just based on whether there exist one or more randomised trials (eg, levels of evidence A–C by international societies), but that the internal and external validities of the trials are quantified against standardised criteria leading to a quality score. This assessment should be standardised across guidance for medicines and technologies. After all, the service user relies on medical technologies and medicines, so why should regulatory standards systematically differ? Should HTA bodies adopt this approach then sponsors of trials of nonpharmaceutical interventions are likely to positively respond with improvements in trial design in order to secure the strongest possible practice guideline recommendation. Adoption of bias minimisation procedures (eg, sham controls) into the design of technology trials will drive improvements in the quality of evidence that subsequently becomes available to HTA reviewers.

## A guideline to rate the quality of evidence and related practice guideline recommendations

We propose a quality of evidence guideline for adoption by stakeholders in clinical research including investigators, sponsors, medical journals, HTA organisations, professional societies and competent authorities. The guideline is based on a tool to identify and quantify evidence impairment through bias and then to objectively assess the quality of evidence, quantified in the score, to rate the strength of a practice guideline recommendation (table 4). The validity, reliability and inter-rater agreement should be assessed and the content optimised through prospective testing.

The questionnaire may be completed by the chief investigator, sponsor or independent reviewers. Considering respondents completing the bias assessment tool, responses from investigators may differ to those of independent, expert reviewers. The option of rating by independent assessors creates the possibility of a reference panel and consensus responses could be used to identify and quantify respondent bias. The validity and utility of this tool merit prospective assessment. As a guide, evidence of significant impairment may be represented by a trial with an open-label design and unmasked ascertainment of the primary outcome. A reviewing organisation may take an executive decision to increase the weighting of a score in exceptional circumstances, such as in the case of scientific misconduct. We believe the data should be made publicly available to inform healthcare providers and service users.

## Turnover of clinical evidence

A second challenge for HTA organisations is the rapidly changing landscape of cardiovascular healthcare technologies and related trials. Clinical trials involving healthcare technologies are less stringently regulated than clinical trials of investigational medicinal products (CTIMPs). There are issues with how to blind and randomise devices and technology (eg, trial of implantable cardioverter defibrillators). This context is conducive to enabling the delivery of technology trials within shorter timescales, and more rapid turnover to the next trial than could be the case for new medicines through CTIMPs. However, there is also a risk with devices inheriting approval status from historical products. Certain healthcare technologies and devices have obtained market authorisation by using data of existing products on the market to substantiate safety and effectiveness data on these newer devices.

The turnover of evidence is externally relevant since a recommendation by a HTA organisation to implement a new technology (or technology-based clinical strategy) may have profound implications for healthcare. Take the CG95 guideline update (November 2016).^2^ Implementing this recommendation in a NHS hospital requires a Radiology Department with highly trained multidisciplinary staff, a multidetector CT scanner with cardiac software and effective clinical administration systems. The NHS lacks the resources to implement the relevant changes in a uniform and timely manner.^28 29^ In the UK, a twofold to fivefold increase in CT coronary angiography will be needed per year to implement the NICE CG95.^2^ In a recent survey, 22 of 70 UK regions had no identifiable accredited CT coronary angiography practitioners.^29^ Advanced imaging with CT coronary angiography, cardiovascular MRI and positron emission tomography involve substantial infrastructure and specialist staff. New guidelines that recommend an increase in adoption of such technologies, such as NICE CG95, have high absolute costs and necessitate substantial resources to be allocated. Trained staff are needed and a lack of staff (cardiologists, radiologists, radiographers) may be the factor that limits adoption. Implementing technology guidelines may take several years to be effected by healthcare providers during which time new trials may have reported leading to changes in the clinical evidence (table 3). This issue is much more relevant to advanced technologies than medicines and becomes problematic if the quality of evidence is impaired by suboptimal trial design and bias. HTA organisations and healthcare providers should take account of this changing landscape. Regular guideline updates are important to take account of new trials and evidence.

## External validity and access to innovative cardiovascular technologies

There may be imbalances in the representation of women and ethnic groups in clinical trials. Such imbalances are externally relevant. In line with the Equality Act 2010, the impact of guidance on protected groups is considered by NICE at the guidance scoping and development stages, and an Equality Impact Assessment is published for each piece of guidance.

The geographic variations in population demographics may limit the generalisability and relevance of a guideline recommendation by a HTA organisation, notably to women and ethnic groups. The availability of expensive healthcare technologies may be associated with socioeconomic and geographic factors, potentially promoting inequality in access to new standards of care in the NHS.

## A global perspective on health technology assessments

Medical technologies for cardiovascular healthcare are often associated with high absolute costs and their availability is influenced by economics. In October 2015, the WHO published a global survey of responses on national HTA systems.^30^ The survey was intended to underpin and inform the aims of universal health coverage. Of 194 member states, 125 nominated national agencies and 111 responded. They were drawn from all six WHO regions. Total healthcare expenditure per capita (US$, 2013) ranged from $17 per person in Eritrea (population 5.23 million) to $9715 per person in Norway (population 5.21 million). There were marked differences in HTA systems including governance, purpose, process transparency and public communication. Germany, the Republic of Korea, Kazakhstan and Romania were the only countries that had these factors in place, along with mandatory involvement of HTA in healthcare decision-making. In comparison, Iceland, Monaco, Qatar and Slovenia, which are all high-income countries, had incomplete HTA systems (across a range of categories), whereas Ecuador ($431 total healthcare expenditure per capita; population 16.14 million), with <1/4 healthcare expenditure and a population >eightfold larger, had an HTA system which fulfilled nearly all of the WHO criteria. The WHO report does not address the issues of bias and impairment of clinical evidence.

## Conclusions

HTA organisations and regulatory bodies should be drivers for improvement in the quality of medical evidence. To this end, these organisations should rate the quality of medical evidence, quantify bias and scale the strength of guideline recommendations accordingly. Improvements in the design and conduct of trials of emergent cardiovascular technologies would likely follow. The rapidly changing landscape of cardiovascular healthcare technologies and related trials (table 3) presents a challenge to HTA organisations and healthcare providers. The turnover of evidence is externally relevant since a recommendation from a HTA organisation to implement a new technology (or related clinical strategy) may have profound implications for providers. Socioeconomic factors, notably for population subgroups, may promote inequalities relating to new standards of care.
